# Japanese fathers’ work-related factors associated with involvement in childcare

**DOI:** 10.1093/joccuh/uiae036

**Published:** 2024-07-15

**Authors:** Manami Ochi, Tsuguhiko Kato, Yuko Kachi, Bibha Dhungel, Mako Nagayoshi, Yuichi Ichinose, Kenji Takehara

**Affiliations:** Department of Health Policy, National Center for Child Health and Development, 2-10-1 Okura, Setagaya-ku, Tokyo 157-8535, Japan; Department of Social Medicine, National Center for Child Health and Development, 2-10-1 Okura, Setagaya-ku, Tokyo 157-8535, Japan; Department of Social Medicine, National Center for Child Health and Development, 2-10-1 Okura, Setagaya-ku, Tokyo 157-8535, Japan; Department of Health Policy, National Center for Child Health and Development, 2-10-1 Okura, Setagaya-ku, Tokyo 157-8535, Japan; School of Psychological Sciences, The University of Melbourne, 12th floor Redmond Barry Building, Parkville Campus, Melbourne, VIC 3052, Australia; Department of Preventive Medicine, Nagoya University Graduate School of Medicine, 65 Tsurumai-cho, Showa-ku, Aichi 466-8550, Japan; Division of Health Services Research, Institute for Cancer Control, National Cancer Center, 5-1-1 Tsukiji, Chuo-ku, Tokyo, 104-0045, Japan; Department of Health Policy, National Center for Child Health and Development, 2-10-1 Okura, Setagaya-ku, Tokyo 157-8535, Japan

**Keywords:** fathers, child-rearing involvement, working mother, work-related environment, Japan

## Abstract

**Objectives:**

Existing studies of fathers’ involvement in childcare have focused on its impact on children’s psychosocial development and the facilitation of family functions, like marital relationships. In this study, we investigated the factors that determine paternal childcare in Japan, particularly focusing on work-related hours and environment, separately, according to mothers’ employment status.

**Methods:**

We used data from the Longitudinal Survey of Newborns in the 21st Century (2010 cohort) conducted in Japan. We restricted the sample to 27 783 participants with working fathers and analyzed how paternal work-related factors affect fathers’ childcare involvement by mothers’ employment status using an ordered logistic regression model.

**Results:**

In the model adjusting for all covariates, the odds ratio (OR) of spending less time with children on weekdays was higher: for fathers who worked 50 and more hours per week compared with those who worked 40-49 hours per week (OR = 1.95, 95% CI: 1.72-2.20 for 50-59 hours), for fathers whose commuting hours were longer than those commuting less than 0.5 hours per day (OR = 2.93, 95% CI: 2.34-3.69 for 1.5 or more hours), for larger workplace employee sizes than for 5-99 employee sizes (OR = 1.56, 95% CI: 1.38-1.77 for 500 or more employees). The associations between these paternal work-related variables and paternal hours spent with the children on weekdays were almost the same if the mothers were working or not working.

**Conclusions:**

Regardless of whether the mother is working, fathers’ work environment factors, such as working hours, play a key role in their involvement in childcare.

## Key points

- **What is already known on this topic:** Fathers’ involvement in childcare, which affects children’s psychosocial development, is known to be determined by factors including work-related factors However, few studies have examined the impact of their working hours on parenting, considering whether mothers are working or not.
- **What this study adds:** In Japan, fathers with long working hours spend less time with their children and engage less frequently in childcare. Association between fathers’ work-related factors and childcare did not change regardless of the maternal employment.
- **How this study might affect research, practice, or policy:** The current situation in which fathers’ work-related hours interfere with their time at home will need to be changed to realize more active father’s involvement in childcare.

## 1. Background

Child-rearing by fathers during infancy plays an important role in the psychosocial development of children and formation of good marital relationships.^1-3^ Early involvement of fathers in child-rearing is particularly important because it is related to their attitudes toward it in later years, such as with school-age children and adolescents.^4-6^ When considering an environment that supports the healthy growth and development of children, it is important to ensure that fathers have sufficient time to care for their children.

Various factors affecting paternal involvement in childcare during infancy have been reported by different studies. Many have mainly examined individual factors (ie, fathering identity and gender ideology), family factors (ie, number of adults, age of youngest child, parents’ education, and employment), and work-related factors (ie, work environment, job autonomy, and working hours).^7-9^ In countries like Japan, where there is a strong sense of gender role division, with fathers as primary breadwinners and mothers responsible for child-rearing, it has been believed that individual factors like gender ideology and fatherhood mainly determine how fathers are involved in child-rearing.^10^ In fact, a recent survey showed that the female/male ratio of hours of unpaid work in Japan is 5.5, which is larger than the average for OECD countries (1.9); and it has been noted that the average time spent by Japanese fathers on childcare is shorter than that of mothers (49 vs 225 min/d).^11^^,^^12^ However, it has also been reported in Japan that the percentage of fathers who want to be more involved in infant and toddler care has increased over time, with the percentage increasing in the younger generation.^13^^,^^14^ Therefore, it is possible that individual factors like gender role division, which explain the difference in parenting time between fathers and mothers in Japan, have changed, and their influence on fathers’ involvement in childcare has decreased. Furthermore, there may be an operational gap between fathers’ desire to take care of their children and their actual allocation of time.^10^

Previous studies have treated fathers’ work-related time as an objectively measurable indicator of their involvement in childcare. Although many studies have reported inverse associations between fathers’ work-related time and childcare involvement,^8^^,^^15-19^ they have been inconsistent regarding the strength of the association. Moreover, in Japan, Ishii-Kuntz^7^ reported that fathers’ working hours and commuting time, together considered as “time availability,” are important factors in their involvement in childcare; however, the study treated “time availability” as a continuous value; as such, it was hard to know how much fathers’ work-related time could prevent them from involvement in childcare. A more detailed examination of fathers’ working hours could reveal the actual impact of fathers’ parenting time. However, fathers’ time for childcare is naturally affected by “family factors” such as whether other family members, such as mothers and grandparents, can be involved in childcare. Particularly in recent years, when the number of working mothers has increased, factors that define fathers’ time availability may depend on their working hours. However, few studies have examined the impact of their working hours on parenting, considering whether mothers are working or not.

In this study, we investigated the factors that determine fathers’ time in childcare in Japan, with a particular focus on factors related to working hours and their working environment, depending on whether the mother is employed.

## 2. Methods

### 2.1. Study sample

**Figure 1:**
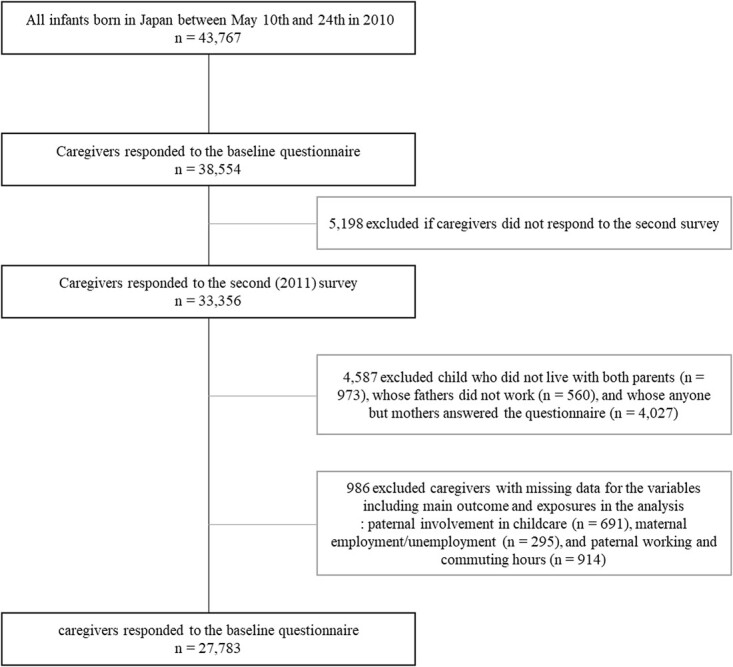
Flow chart for study samples.

We used data from the Longitudinal Survey of Newborns in the 21st Century (2010 cohort), a population-based survey conducted by the Ministry of Health, Labor, and Welfare in Japan. The study sample included all infants born in Japan between May 10 and 24, 2010, using birth records from the national vital statistics. The baseline survey was mailed to parents when their infants were 6 months old (*n* = 43 767). A total of 38 554 caregivers responded to the baseline questionnaire (response rate, 88.1%). After the baseline survey, annual surveys were administered via mail to participants. We used data from the 2010 and 2011 waves of the survey. In total, 33 356 caregivers (86.5 %) responded to the 2011 survey. For our study, we included children who lived with both parents, whose fathers had jobs, and those whose mothers answered the questionnaire to maintain consistency in the assessment of childcare (excluded samples *n* = 4587, 13.8%). We excluded responses with missing data for the variables including the main outcome and exposures in the analysis: paternal involvement in childcare (*n* = 691, 2.5%) and maternal employment/unemployment (*n* = 295, 1.1%). Finally, data of 27 783 newborns were included in the analysis (83.3% of respondents in the 2011 survey wave; Figure 1).

### 2.2. Exposure

We selected the following variables as work-related factors to determine paternal childcare: paternal working hours per week, paternal commuting hours per day, and the number of employees at the paternal workplace. Working hours per week was ascertained by the following answer options: <20 hours, 20-39 hours, 40-49 hours, 50-59 hours, and ≥60 hours. Commuting hours per day was ascertained by the following answer options: none, 0.1-0.49 hours, 0.5-0.9 hours, 1-1.49 hours, and ≥1.5 hours. We merged the no-commute category and the less than 0.5 hours category. The questionnaire also asked employed fathers about the number of employees at their workplace, using the following options: 1-4, 5-99, 100-499, ≥500, and government administration office.

### 2.3. Outcome: paternal childcare

Paternal childcare was assessed at 18 months of age using the following 3 measures: paternal childcare hours on working days, paternal childcare hours on nonworking days, and the frequency of each type of caregiving (feeding, diaper change, bathing, putting the child to sleep, playing at home, and taking the child outside).

Paternal childcare hours were ascertained using the following questions: “How much time does the father spend with his child in a day, on average, except for sleeping hours?” Respondents answered about both working days and nonworking days from the following options: none, <0.5 hours, 0.5-0.9 hours, 1-1.9 hours, 2-3.9 hours, 4-5.9 hours, and ≥6 hours. Thereafter, we defined categories, making sure to evenly distribute in the following new categories: “<0.5 hours,” “0.5-1.9 hours,” and “≥2 hours” for working days, and “<4 hours,” “4-5.9 hours,” and “≥6 hours” for nonworking days. Frequency of each type of paternal caregiving was retrieved by 6 items: (1) feeding, (2) changing diaper, (3) bathing, (4) putting the child to sleep, (5) playing with the child at home, and (6) taking the child outside. Responses to each question included “not at all,” “rarely,” “sometimes,” and “always.” To calculate the total paternal caregiving scores, each response was scored ranging from 0 to 3 (ie, “not at all” = 0; and “always” = 3). The measurement and method of specifying the caregiving variable were in line with those of previous studies.^20^^,^^21^ Thus, the total paternal caregiving score ranged from 0 to 18 (Cronbach a = .80). We divided the scores equally into 3 groups based on the previous study^22^: low (0-6), medium (7-12), and high (13-18).

### 2.4. Covariates

For this study, we chose the following variables that could influence fathers’ involvement in childcare as individual and family domain factors^5^^,^^23^: number of siblings, living with grandparent(s), paternal and maternal age at childbirth (<25, 25-29, 30-34, 35-39, ≥40), paternal and maternal education (lower than high-school degree, high-school degree, some college degree, and college degree or more), annual household income (in Japanese yen, JPY) at 2010 (<4 million, 4-5.9 million, 6-7.9 million, 8-9.9 million, ≥10 million), sex of the child, birth weight (<500 g or ≥2500 g), and child’s history of hospital admission or visits for congenital diseases. For income, equivalent household income (JPY) was calculated by adjusting for the square root of the number of persons living in the household and then categorized into 5 categories (<2.5 million, 2.5-3.9 million, 4.0-5.4 million, 5.5-6.9 million, ≥7.0 million).

### 2.5. Statistical analysis

We developed an ordered logistic regression model; and the odds ratios (ORs) and 95% CIs were calculated by adjusting for the covariates shown above. In addition to the crude models, we adjusted for paternal work-related factors and covariates to assess their impact on fathers’ involvement in childcare. To compare whether factors related to paternal childcare differed between dual-earner and single-earner households, we analyzed the data according to whether the mother was working when their child was 1.5 years old. We also tested how the interaction term between mother’s working status and father’s work-related factors (working hours, commuting hours, and the number of employees at father’s workplace) influenced the outcomes (father’s childcare). We analyzed only the complete cases.

Analyses were performed using Stata software (version 17.0; Stata Corp., College Station, TX, USA). Responses to questionnaires from caregivers were considered as consent to participate in the study; thus, written or verbal consent was not required in the data collection process. This study was approved by the National Center for Child Health and Development Ethics Committee (No. 2020-299).

## 3. Results

Table 1 presents the characteristics of the respondents according to their working status. Fathers working 40 hours or more per week accounted for 87.3% and 86.4% of those in working-mother and in non–working-mother households. Fathers who commuted for more than 1 hour per day accounted for 14.3% and 15.7%, in working-mother and in non–working-mother households, respectively. Fathers working in organizations with more than 500 employees accounted for 22.9% and 31.2% of the samples, in the above-mentioned types of households, respectively. Fathers who spent less than 0.5 hours with their children on working days accounted for 5.1% of working-mother households and 7.9% of non–working-mother households. Fathers who spent less than 4 hours with their children on nonworking days accounted for 11% and 13.6%, in each kind of household, respectively. The groups in which fathers spent the least amount of time caring for their children (0-6 points) accounted for 6.2% and 8.9%, respectively. Paternal caregiving in one type of activity was not strongly correlated with another type of activity (Spearman correlation coefficients range, 0.27-0.50, *P* values for all tests were <.001; Table 2).

**Table 1:** Characteristics of the study participants, stratified by maternal working or not.

Characteristic		Working mother *n* = 11 615		Nonworking mother *n* = 16 168		Total *n* = 27 783	
		No. (Mean)	% (SD)	No. (Mean)	% (SD)	No. (Mean)	% (SD)
Father’s age at childbirth		(33.0)	(5.4)	(32.8)	(5.5)	(32.9)	(5.4)
Mother’s age at childbirth		(31.3)	(4.6)	(31.0)	(4.7)	(31.1)	(4.7)
Paternal education	Less than high school	835	7.2	1015	6.3	1850	6.7
	High school	3670	31.6	4947	30.6	8617	31.0
	Some college	2304	19.8	2826	17.5	5130	18.5
	College or higher	4775	41.1	7346	45.4	12 121	43.6
	Missing	31	0.3	34	0.2	65	0.2
Maternal education	Less than high school	396	3.4	778	4.8	1174	4.2
	High school	2761	23.8	4740	29.3	7501	27.0
	Some college	4826	41.5	6790	42.0	11 616	41.8
	College or higher	3617	31.1	3844	23.8	7461	26.9
	Missing	15	0.1	16	0.1	31	0.1
Paternal working hours per week	<20	417	3.6	740	4.6	1157	4.2
	20-39	766	6.6	896	5.5	1662	6.0
	40-49	3949	34.0	5053	31.3	9002	32.4
	50-59	3134	27.0	4481	27.7	7615	27.4
	≥ 60	3056	26.3	4429	27.4	7485	26.9
	Missing	293	2.5	569	3.5	862	3.1
Paternal commuting hours per day	<0.5	6207	53.4	8073	49.9	14 280	51.4
	0.5-0.9	3294	28.4	4767	29.5	8061	29.0
	1-1.49	1267	10.9	1995	12.3	3262	11.7
	≥1.5	400	3.4	551	3.4	951	3.4
	Missing	447	3.8	782	4.8	1229	4.4
The number of employees at father’s workplace	1-4	417	3.6	428	2.6	845	3.0
	5-99	3484	30.0	4365	27.0	7849	28.3
	100-499	2392	20.6	3325	20.6	5717	20.6
	≥500	2656	22.9	5050	31.2	7706	27.7
	Government office	870	7.5	1003	6.2	1873	6.7
Child sex	Boy	5988	51.6	8255	51.1	14 243	51.3
	Girl	5627	48.4	7913	48.9	13 540	48.7
Birth weight	≥2500 g	10 649	91.7	14 621	90.4	25 270	91.0
	<2500 g	966	8.3	1545	9.6	2511	9.0
	Missing	0	0	2	0.0	2	0
Child’s history of hospital visits for congenital diseases	No	11 102	95.6	15 152	93.7	26 254	94.5
	Yes	222	1.9	321	2.0	543	2.0
	Missing	291	2.5	695	4.3	986	3.5
Child’s history of hospital admission for congenital diseases	No	11 235	96.7	15 346	94.9	26 581	95.7
	Yes	89	0.8	127	0.8	216	0.8
	Missing	291	2.5	695	4.3	986	3.5
Number of siblings	Only child	5302	45.6	7753	48.0	13 055	47.0
	1	4393	37.8	6240	38.6	10 633	38.3
	≥2	1920	16.5	2175	13.5	4095	14.7
Equivalent household income (million JPY)	<2.5	3158	27.2	5313	32.9	8471	30.5
	2.5-3.9	3415	29.4	6097	37.7	9512	34.2
	4.0-5.4	2393	20.6	2658	16.4	5051	18.2
	5.5-6.9	1315	11.3	895	5.5	2210	8.0
	≥7.0	973	8.4	574	3.6	1547	5.6
	Missing	361	3.1	631	3.9	992	3.6
Living with grandparents	No	9539	82.1	13 879	85.8	23 418	84.3
	Yes	2076	17.9	2289	14.2	4365	15.7
Paternal childcare hours on working days	<0.5	588	5.1	1275	7.9	1863	6.7
	0.5-1.9	2776	23.9	5462	33.8	8238	29.7
	≥2	8251	71.0	9431	58.3	17 682	63.6
Paternal childcare hours on nonworking days	≥6	9270	79.8	12 194	75.4	21 464	77.3
	4-5.9	1067	9.2	1776	11.0	2843	10.2
	<4	1278	11.0	2198	13.6	3476	12.5
Frequency of each type of caregiving (pt.)	13-18	5145	44.3	4515	27.9	9660	34.8
	7-12	5755	49.5	10 207	63.1	15 962	57.5
	0-6	715	6.2	1446	8.9	2161	7.8

Abbreviations: JPY, Japanese yen; pt., point

**Table 2:** Spearman correlation coefficients between specific types of paternal involvement in caregiving.^a^

	(1)	(2)	(3)	(4)	(5)	(6)
(1) Feeding	1.00					
(2) Changing diaper	0.50	1.00				
(3) Bathing	0.35	0.31	1.00			
(4) Putting the child to sleep	0.42	0.42	0.40	1.00		
(5) Playing with the child at home	0.35	0.31	0.36	0.30	1.00	
(6) Taking the child outside	0.32	0.30	0.27	0.28	0.44	1.00

a *P* values for all tests <.001.

Table 3 shows the results of crude and multiple ordered logistic regressions, analyzing the association between paternal work-related factors and the risk of shorter paternal childcare hours on working days. In the model adjusting for all covariates, the OR of spending less time with children on working days was higher for fathers who worked for 50 and more hours per week compared with those who worked 40-50 hours per week in the group where mothers were employed (OR = 1.95, 95% CI: 1.72-2.20 for 50-59 hours and OR = 3.78, 95% CI: 3.35-4.26 for 60 and more hours). Moreover, fathers who worked less than 20 hours per week had a higher OR of spending less time with their children on working days compared with those who worked 40-50 hours per week (OR = 1.63, 95% CI: 1.20-2.22). Compared with fathers commuting for less than 0.5 hours, the OR of fathers who spent less time with their children on working days was higher the greater the father’s commuting hours (OR = 2.31, 95% CI: 2.00-2.66 for 1-1.5 hours; OR = 2.93, 95% CI: 2.34-3.69 for ≥1.5 hours). The OR of fathers spending less time with their children on working days was higher for those with a larger workplace employee size than for 5-99 employee sizes (OR = 1.27, 95% CI: 1.11-1.44 for 100-499 employees; OR = 1.56, 95% CI: 1.38-1.77 for ≥500 employees). The associations between these 3 paternal work-related variables and the amount of time that fathers spent with their children on working days were almost the same for families where mothers were not working.

**Table 3:** Crude and adjusted ordered logistic regression of the associations between paternal work-related factors and the risk of shorter paternal childcare hours on working days.^a^

	**Working mother**						**Nonworking mother**					
	**Crude**			**Adjusted model** ^**b**^			**Crude**			**Adjusted model** ^**b**^		
	**OR**	**(95% CI)**		**OR**	**(95% CI)**		**OR**	**(95% CI)**		**OR**	**(95% CI)**	
**Paternal working hours per week**												
**<20**	1.16	0.91	1.49	**1.63**	**1.20**	**2.22**	1.16	0.98	1.36	**1.75**	**1.41**	**2.16**
**20-39**	**0.80**	**0.65**	**0.99**	0.86	0.67	1.09	**0.81**	**0.69**	**0.95**	0.90	0.74	1.08
**40-49**	(reference)			(reference)			(reference)			(reference)		
**50-59**	**1.92**	**1.72**	**2.14**	**1.95**	**1.72**	**2.20**	**1.92**	**1.77**	**2.09**	**1.95**	**1.77**	**2.14**
**≥60**	**3.39**	**3.05**	**3.77**	**3.78**	**3.35**	**4.26**	**3.12**	**2.88**	**3.39**	**3.46**	**3.15**	**3.80**
**Paternal commuting hours per day**												
**<0.5**	(reference)			(reference)			(reference)			(reference)		
**0.5-0.9**	**1.53**	**1.40**	**1.68**	**1.36**	**1.22**	**1.52**	**1.66**	**1.55**	**1.78**	**1.45**	**1.34**	**1.58**
**1-1.49**	**2.56**	**2.26**	**2.90**	**2.31**	**2.00**	**2.66**	**2.27**	**2.07**	**2.50**	**2.04**	**1.83**	**2.28**
**≥1.5**	**3.13**	**2.56**	**3.82**	**2.93**	**2.34**	**3.69**	**2.79**	**2.36**	**3.30**	**2.63**	**2.18**	**3.18**
**Paternal workplace headcount size**												
**1-4**	**0.74**	**0.58**	**0.95**	0.77	0.59	1.01	**0.51**	**0.40**	**0.64**	**0.56**	**0.43**	**0.73**
**5-99**	(reference)			(reference)			(reference)			(reference)		
**100-499**	**1.25**	**1.11**	**1.40**	**1.27**	**1.11**	**1.44**	**1.40**	**1.29**	**1.54**	**1.26**	**1.13**	**1.39**
**≥500**	**1.71**	**1.53**	**1.90**	**1.56**	**1.38**	**1.77**	**1.87**	**1.72**	**2.02**	**1.49**	**1.36**	**1.64**
**Government office**	**0.80**	**0.67**	**0.96**	**0.74**	**0.61**	**0.90**	1.05	0.92	1.21	0.86	0.73	1.01
**Father’s age at birth**												
**<25**	(reference)			(reference)			(reference)			(reference)		
**25-29**	**1.42**	**1.13**	**1.79**	0.99	0.72	1.35	**1.69**	**1.44**	**1.99**	1.07	0.86	1.34
**30-34**	**1.67**	**1.33**	**2.09**	1.05	0.76	1.46	**2.35**	**2.00**	**2.75**	1.20	0.95	1.52
**35-39**	**1.78**	**1.42**	**2.24**	1.10	0.79	1.55	**2.50**	**2.13**	**2.93**	1.15	0.90	1.46
**40+**	**1.51**	**1.18**	**1.94**	0.97	0.67	1.40	**2.17**	**1.82**	**2.59**	0.93	0.71	1.21
**Mother’s age at birth**												
**<25**	(reference)			(reference)			(reference)			(reference)		
**25-29**	**1.43**	**1.19**	**1.73**	1.18	0.90	1.54	**1.61**	**1.41**	**1.83**	1.12	0.93	1.34
**30-34**	**1.73**	**1.44**	**2.08**	1.27	0.96	1.68	**2.15**	**1.90**	**2.44**	**1.24**	**1.02**	**1.51**
**35-39**	**1.62**	**1.34**	**1.96**	1.15	0.85	1.54	**2.27**	**1.99**	**2.59**	**1.27**	**1.03**	**1.56**
**40+**	**1.80**	**1.38**	**2.35**	1.44	0.98	2.11	**2.13**	**1.73**	**2.62**	**1.44**	**1.08**	**1.93**
**Paternal education**												
**Less than high school**	(reference)			(reference)			(reference)			(reference)		
**High school**	0.91	0.76	1.08	**0.75**	**0.60**	**0.93**	**1.17**	**1.01**	**1.36**	0.96	0.79	1.17
**Some college**	1.05	0.87	1.26	0.85	0.67	1.08	**1.69**	**1.45**	**1.97**	**1.30**	**1.06**	**1.60**
**College or higher**	**1.55**	**1.31**	**1.83**	0.98	0.78	1.23	**2.59**	**2.25**	**2.99**	**1.58**	**1.29**	**1.93**
**Maternal education**												
**Less than high school**	(reference)			(reference)			(reference)			(reference)		
**High school**	1.18	0.92	1.53	1.18	0.86	1.62	**1.26**	**1.07**	**1.49**	1.22	0.99	1.52
**Some college**	**1.29**	**1.01**	**1.66**	1.10	0.80	1.51	**1.78**	**1.52**	**2.09**	**1.40**	**1.13**	**1.73**
**College or higher**	**1.80**	**1.40**	**2.31**	1.30	0.94	1.80	**2.27**	**1.92**	**2.68**	**1.46**	**1.17**	**1.82**
**Child sex**												
**Boy**	(reference)			(reference)			(reference)			(reference)		
**Girl**	0.99	0.91	1.07	1.01	0.92	1.10	1.04	0.98	1.10	1.06	0.98	1.14
**Birth weight**												
**≥2500 g**	(reference)			(reference)			(reference)			(reference)		
**<2500 g**	0.98	0.85	1.13	0.97	0.82	1.16	0.99	0.89	1.10	1.03	0.91	1.16
**Child’s history of hospital visits for congenital diseases**												
**No**	(reference)			(reference)			(reference)			(reference)		
**Yes**	0.83	0.61	1.12	1.09	0.70	1.69	0.89	0.71	1.11	0.72	0.52	1.00
**Child’s history of hospital admission for congenital diseases**												
**No**	(reference)			(reference)			(reference)			(reference)		
**Yes**	0.80	0.49	1.31	0.78	0.38	1.60	0.78	0.55	1.12	1.01	0.60	1.71
**Number of siblings**												
**Only child**	(reference)			(reference)			(reference)			(reference)		
**1**	**1.16**	**1.06**	**1.26**	**1.30**	**1.16**	**1.45**	**1.43**	**1.34**	**1.53**	**1.55**	**1.42**	**1.69**
**≥2**	1.11	0.99	1.24	**1.42**	**1.22**	**1.66**	**1.33**	**1.21**	**1.46**	**1.58**	**1.40**	**1.79**
**Equivalent income (million yen)**												
**<250**	(reference)			(reference)			(reference)			(reference)		
**250-399**	**1.26**	**1.13**	**1.41**	1.11	0.97	1.28	**1.42**	**1.32**	**1.53**	**1.15**	**1.04**	**1.27**
**400-549**	**1.29**	**1.15**	**1.46**	1.11	0.94	1.31	**1.71**	**1.56**	**1.88**	**1.20**	**1.06**	**1.37**
**550-699**	**1.56**	**1.35**	**1.79**	1.20	0.98	1.46	**1.96**	**1.71**	**2.24**	**1.28**	**1.07**	**1.52**
**700+**	**1.89**	**1.63**	**2.20**	1.18	0.95	1.49	**2.06**	**1.74**	**2.42**	**1.29**	**1.04**	**1.59**
**Living with grandparents**												
**No**	(reference)			(reference)			(reference)			(reference)		
**Yes**	0.96	0.87	1.07	**1.34**	**1.17**	**1.53**	**0.85**	**0.77**	**0.92**	**1.31**	**1.17**	**1.47**

Abbreviation: OR, odds ratio.

a Bold values denote statistical significance at *P* < .05.

b Adjusted model includes paternal work-related factors (paternal working hours per week, paternal commuting hours per day, paternal workplace headcount size) and covariates (father’s age at birth, mother’s age at birth, paternal education, maternal education, child sex, birth weight, child’s history of hospital visits for congenital diseases, child’s history of hospital admission for congenital diseases, number of siblings, equivalent income, living with grandparents).

Table 4 shows the results of the crude and adjusted ordered logistic regression analyses of the association between paternal work-related factors and the risk of shorter paternal childcare hours on nonworking days. In the model adjusting for all covariates, the OR of spending less time with children on nonworking days was higher for fathers who worked more than 50 hours per week compared with fathers who worked 40-49 hours per week in the group where mothers were employed (OR = 1.18, 95% CI: 1.03-1.36 for 50-59 hours and OR = 1.46, 95% CI: 1.28-1.68 for ≥60 hours). Furthermore, fathers who worked for less than 20 hours per week had a higher OR of spending less time with their children on nonworking days than fathers who worked 40-49 hours per week (OR = 1.46, 95% CI: 1.06-1.99). Fathers who commuted for 1-1.49 hours had a lower OR of spending less time with their children on nonworking days than those who commuted less than 0.5 hours to work (OR = 0.82, 95% CI: 0.68-0.98). We did not observe an association between the number of fathers’ workplace employees and the amount of time spent with their children on nonworking days. The associations between these 3 paternal work-related variables and the time fathers spent with their children on nonworking days were similar for families where mothers were not working. However, in families where fathers worked 20 to 39 hours per week and mothers were not working, there was a higher risk of fathers spending less time with their children on nonworking days compared with those where fathers worked 40 hours. This trend differed from families where mothers were working.

**Table 4:** Crude and adjusted ordered logistic regression of the associations between fathers’ work-related factors and the risk of shorter paternal childcare hours on nonworking days.^a^

	**Working mother**						**Nonworking mother**					
	**Crude**			**Adjusted model** ^**b**^			**Crude**			**Adjusted model** ^**b**^		
	**OR**	**(95% CI)**		**OR**	**(95% CI)**		**OR**	**(95% CI)**		**OR**	**(95% CI)**	
**Paternal working hours per week**												
**<20**	**1.51**	**1.19**	**1.91**	**1.46**	**1.06**	**1.99**	1.09	0.91	1.31	1.21	0.95	1.53
**20-39**	1.19	0.98	1.45	1.05	0.83	1.33	**1.32**	**1.13**	**1.54**	**1.35**	**1.13**	**1.63**
**40-49**	(reference)			(reference)			(reference)			(reference)		
**50-59**	**1.16**	**1.03**	**1.31**	**1.18**	**1.03**	**1.36**	1.06	0.97	1.17	**1.13**	**1.02**	**1.26**
**≥60**	**1.46**	**1.30**	**1.64**	**1.46**	**1.28**	**1.68**	**1.24**	**1.13**	**1.36**	**1.31**	**1.18**	**1.46**
**Paternal commuting hours per day**												
**<0.5**	(reference)			(reference)			(reference)			(reference)		
**0.5-0.9**	**0.83**	**0.75**	**0.92**	0.91	0.80	1.03	**0.83**	**0.76**	**0.90**	**0.80**	**0.73**	**0.88**
**1-1.49**	**0.69**	**0.59**	**0.82**	**0.82**	**0.68**	**0.98**	0.94	0.84	1.05	0.90	0.80	1.03
**≥1.5**	0.98	0.76	1.25	0.95	0.71	1.28	0.84	0.69	1.03	0.82	0.65	1.04
**Paternal workplace headcount size**												
**1-4**	0.89	0.68	1.15	0.83	0.63	1.10	0.91	0.72	1.16	0.85	0.66	1.11
**5-99**	(reference)			(reference)			(reference)			(reference)		
**100-499**	0.93	0.81	1.05	1.04	0.90	1.20	1.08	0.97	1.20	1.09	0.97	1.22
**≥500**	**0.74**	**0.65**	**0.84**	0.89	0.76	1.03	1.01	0.92	1.11	1.06	0.95	1.19
**Government office**	0.85	0.70	1.03	0.99	0.80	1.22	1.05	0.89	1.23	1.03	0.86	1.23
**Father’s age at birth**												
**<25**	(reference)			(reference)			(reference)			(reference)		
**25-29**	0.82	0.65	1.05	0.73	0.53	1.02	0.97	0.81	1.16	1.06	0.82	1.37
**30-34**	1.00	0.80	1.27	0.78	0.56	1.10	**1.23**	**1.03**	**1.46**	1.11	0.85	1.44
**35-39**	**1.36**	**1.08**	**1.72**	0.96	0.67	1.36	**1.67**	**1.40**	**1.99**	**1.37**	**1.05**	**1.81**
**40+**	**1.72**	**1.34**	**2.21**	1.17	0.80	1.71	**1.97**	**1.63**	**2.38**	**1.48**	**1.10**	**1.98**
**Mother’s age at birth**												
**<25**	(reference)			(reference)			(reference)			(reference)		
**25-29**	1.04	0.84	1.28	1.20	0.89	1.62	1.02	0.88	1.18	1.03	0.83	1.27
**30-34**	**1.37**	**1.12**	**1.68**	**1.51**	**1.10**	**2.07**	**1.40**	**1.21**	**1.61**	**1.30**	**1.04**	**1.62**
**35-39**	**1.69**	**1.37**	**2.09**	**1.60**	**1.14**	**2.23**	**1.83**	**1.58**	**2.12**	**1.43**	**1.13**	**1.82**
**40+**	**2.16**	**1.62**	**2.87**	**1.72**	**1.12**	**2.64**	**2.47**	**1.98**	**3.08**	**2.01**	**1.47**	**2.75**
**Paternal education**												
**Less than high school**	(reference)			(reference)			(reference)			(reference)		
**High school**	**0.64**	**0.54**	**0.75**	**0.60**	**0.48**	**0.75**	0.88	0.76	1.02	0.98	0.80	1.20
**Some college**	**0.65**	**0.54**	**0.78**	**0.62**	**0.49**	**0.79**	**0.81**	**0.69**	**0.95**	0.90	0.72	1.12
**College or higher**	**0.58**	**0.49**	**0.69**	**0.56**	**0.44**	**0.71**	**0.83**	**0.71**	**0.96**	0.96	0.78	1.18
**Maternal education**												
**Less than high school**	(reference)			(reference)			(reference)			(reference)		
**High school**	1.05	0.81	1.35	1.32	0.94	1.86	0.94	0.79	1.11	0.90	0.72	1.12
**Some college**	0.86	0.67	1.10	1.26	0.89	1.77	0.92	0.78	1.09	0.86	0.69	1.08
**College or higher**	0.87	0.67	1.12	**1.48**	**1.04**	**2.10**	0.90	0.76	1.07	0.95	0.75	1.20
**Child sex**												
**Boy**	(reference)			(reference)			(reference)			(reference)		
**Girl**	1.03	0.94	1.13	1.01	0.90	1.12	1.01	0.94	1.08	0.98	0.90	1.06
**Birth weight**												
**≥2500 g**	(reference)			(reference)			(reference)			(reference)		
**<2500 g**	1.02	0.87	1.20	0.97	0.80	1.18	**1.23**	**1.10**	**1.39**	1.12	0.98	1.29
**Child’s history of hospital visits for congenital diseases**												
**No**	(reference)			(reference)			(reference)			(reference)		
**Yes**	1.27	0.93	1.74	1.33	0.84	2.12	1.19	0.93	1.52	0.92	0.64	1.33
**Child’s history of hospital admission for congenital diseases**												
**No**	(reference)			(reference)			(reference)			(reference)		
**Yes**	1.37	0.86	2.19	1.03	0.49	2.18	1.25	0.84	1.85	1.16	0.64	2.11
**Number of siblings**												
**Only child**	(reference)			(reference)			(reference)			(reference)		
**1**	**1.26**	**1.14**	**1.39**	1.12	0.98	1.28	**1.31**	**1.21**	**1.42**	**1.13**	**1.03**	**1.25**
**≥2**	**1.93**	**1.71**	**2.18**	**1.57**	**1.33**	**1.86**	**1.97**	**1.78**	**2.18**	**1.55**	**1.36**	**1.77**
**Equivalent income (million yen)**												
**<250**	(reference)			(reference)			(reference)			(reference)		
**250-399**	**0.78**	**0.69**	**0.87**	**0.86**	**0.74**	**0.99**	**0.81**	**0.74**	**0.88**	0.90	0.80	1.00
**400-549**	**0.65**	**0.57**	**0.74**	**0.78**	**0.65**	**0.94**	**0.80**	**0.72**	**0.89**	0.90	0.78	1.04
**550-699**	**0.58**	**0.49**	**0.68**	**0.74**	**0.59**	**0.94**	**0.78**	**0.66**	**0.92**	0.88	0.71	1.08
**700+**	**0.64**	**0.53**	**0.76**	**0.70**	**0.54**	**0.92**	0.91	0.75	1.10	0.87	0.67	1.12
**Living with grandparents**												
**No**	(reference)			(reference)			(reference)			(reference)		
**Yes**	**1.44**	**1.29**	**1.61**	**1.19**	**1.03**	**1.37**	**1.31**	**1.19**	**1.44**	**1.33**	**1.17**	**1.50**

Abbreviation: OR, odds ratio.

a Bold values denote statistical significance at *P* < .05.

b Adjusted model includes paternal work-related factors (paternal working hours per week, paternal commuting hours per day, paternal workplace headcount size) and covariates (father’s age at birth, mother’s age at birth, paternal education, maternal education, child sex, birth weight, child’s history of hospital visits for congenital diseases, child’s history of hospital admission for congenital diseases, number of siblings, equivalent income, living with grandparents).

Table 5 shows the results of the crude and adjusted ordered logistic regression of the association between paternal work-related factors and the risk of reduced frequency of paternal caregiving. In the model adjusting for all covariates, the OR of less frequent paternal childcare was found to be higher for fathers working for more than 50 hours per week than those working for 40-49 hours per week in the working mother group (OR = 1.45, 95% CI: 1.30-1.60 for 50-59 hours and OR = 2.47, 95% CI: 2.22-2.75 for ≥60 hours). Compared with fathers who worked for 40-49 hours per week, those who worked less than 20 hours per week also had higher OR for less frequent childcare (OR = 1.46, 95% CI: 1.13-1.89). It was noted that the OR of less frequent childcare by fathers was higher with longer paternal commuting hours compared with less than 0.5 commuting hours (OR = 1.44, 95% CI: 1.26-1.66 for 1-1.5 hours; OR = 2.01, 95% CI: 1.61-2.51 for ≥1.5 hours). The OR for less frequent paternal childcare was higher for those whose workplace had 500 or more employees than for those whose workplace had 5-99 employees (OR = 1.27, 95% CI: 1.13-1.42). The associations between the 3 fathers’ work-related variables and the frequency of paternal childcare were similar for families where mothers were not working.

**Table 5:** Crude and adjusted ordered logistic regression of the associations between paternal work-related factors and the risk of reduced frequency of paternal caregiving.^a^

	**Working mother**						**Nonworking mother**					
	**Crude**			**Adjusted model** ^**b**^			**Crude**			**Adjusted model** ^**b**^		
	**OR**	**(95% CI)**		**OR**	**(95% CI)**		**OR**	**(95% CI)**		**OR**	**(95% CI)**	
**Paternal working hours per week**												
**<20**	**1.45**	**1.19**	**1.77**	**1.46**	**1.13**	**1.89**	**1.18**	**1.01**	**1.38**	**1.44**	**1.17**	**1.76**
**20-39**	0.90	0.77	1.05	0.91	0.76	1.09	0.98	0.85	1.13	1.03	0.87	1.21
**40-49**	(reference)			(reference)			(reference)			(reference)		
**50-59**	**1.45**	**1.32**	**1.59**	**1.45**	**1.30**	**1.60**	**1.52**	**1.40**	**1.65**	**1.50**	**1.37**	**1.64**
**≥60**	**2.35**	**2.14**	**2.59**	**2.47**	**2.22**	**2.75**	**2.31**	**2.12**	**2.51**	**2.41**	**2.19**	**2.65**
**Paternal commuting hours per day**												
**<0.5**	(reference)			(reference)			(reference)			(reference)		
**0.5-0.9**	**1.11**	**1.02**	**1.20**	**1.17**	**1.06**	**1.29**	**1.17**	**1.09**	**1.26**	1.08	0.99	1.17
**1-1.49**	**1.36**	**1.21**	**1.53**	**1.44**	**1.26**	**1.66**	**1.31**	**1.18**	**1.45**	**1.20**	**1.07**	**1.34**
**≥1.5**	**1.80**	**1.48**	**2.19**	**2.01**	**1.61**	**2.51**	**1.36**	**1.14**	**1.62**	**1.28**	**1.05**	**1.56**
**Paternal workplace headcount size**												
**1-4**	0.94	0.77	1.14	0.89	0.71	1.1	0.87	0.71	1.07	0.87	0.71	1.07
**5-99**	(reference)			(reference)			(reference)			(reference)		
**100-499**	0.98	0.89	1.09	1.05	0.94	1.17	**1.12**	**1.08**	**1.30**	**1.19**	**1.08**	**1.30**
**≥500**	**1.18**	**1.07**	**1.31**	**1.27**	**1.13**	**1.42**	**1.35**	**1.24**	**1.46**	**1.35**	**1.24**	**1.47**
**Government office**	**0.76**	**0.66**	**0.88**	**0.83**	**0.71**	**0.98**	0.88	0.77	1.01	0.88	0.77	1.01
**Father’s age at birth**												
**<25**	(reference)			(reference)			(reference)			(reference)		
**25-29**	0.90	0.75	1.08	0.80	0.62	1.04	1.11	0.96	1.28	0.91	0.75	1.12
**30-34**	1.11	0.93	1.32	0.93	0.72	1.21	**1.48**	**1.28**	**1.70**	1.08	0.87	1.33
**35-39**	**1.22**	**1.02**	**1.46**	1.05	0.80	1.39	**1.68**	**1.46**	**1.95**	1.17	0.94	1.46
**40+**	**1.28**	**1.04**	**1.56**	1.11	0.82	1.49	**1.63**	**1.38**	**1.92**	1.13	0.88	1.45
**Mother’s age at birth**												
**<25**	(reference)			(reference)			(reference)			(reference)		
**25-29**	0.98	0.84	1.14	1.06	0.85	1.32	**1.31**	**1.16**	**1.47**	**1.20**	**1.02**	**1.42**
**30-34**	**1.22**	**1.06**	**1.42**	1.19	0.95	1.50	**1.64**	**1.46**	**1.84**	**1.34**	**1.12**	**1.60**
**35-39**	**1.26**	**1.07**	**1.47**	1.14	0.89	1.46	**1.74**	**1.54**	**1.97**	**1.29**	**1.06**	**1.58**
**40+**	**1.28**	**1.02**	**1.62**	1.13	0.81	1.59	**2.01**	**1.63**	**2.48**	**1.74**	**1.30**	**2.32**
**Paternal education**												
**Less than high school**	(reference)			(reference)			(reference)			(reference)		
**High school**	**0.68**	**0.59**	**0.79**	**0.64**	**0.52**	**0.77**	**0.86**	**0.75**	**0.99**	**0.80**	**0.67**	**0.96**
**Some college**	**0.62**	**0.53**	**0.73**	**0.62**	**0.51**	**0.76**	0.97	0.84	1.13	0.88	0.73	1.07
**College or higher**	**0.78**	**0.68**	**0.91**	**0.73**	**0.59**	**0.89**	**1.22**	**1.06**	**1.39**	1.00	0.83	1.21
**Maternal education**												
**Less than high school**	(reference)			(reference)			(reference)			(reference)		
**High school**	0.96	0.78	1.18	1.13	0.87	1.48	1.06	0.91	1.24	1.09	0.89	1.32
**Some college**	0.82	0.67	1.00	0.99	0.75	1.29	**1.25**	**1.07**	**1.45**	1.17	0.96	1.43
**College or higher**	0.93	0.76	1.14	1.10	0.83	1.45	**1.31**	**1.12**	**1.54**	1.12	0.91	1.37
**Child sex**												
**Boy**	(reference)			(reference)			(reference)			(reference)		
**Girl**	1.07	0.99	1.15	**1.11**	**1.02**	**1.21**	**1.12**	**1.05**	**1.19**	**1.13**	**1.05**	**1.22**
**Birth weight**												
**≥2500 g**	(reference)			(reference)			(reference)			(reference)		
**<2500 g**	0.92	0.81	1.04	**0.84**	**0.72**	**0.97**	0.94	0.84	1.04	0.89	0.78	1.01
**Child’s history of hospital visits for congenital diseases**												
**No**	(reference)			(reference)			(reference)			(reference)		
**Yes**	0.94	0.72	1.22	0.87	0.59	1.29	0.98	0.78	1.23	0.73	0.53	1.00
**Child’s history of hospital admission for congenital diseases**												
**No**	(reference)			(reference)			(reference)			(reference)		
**Yes**	1.15	0.76	1.74	1.60	0.85	3.02	1.27	0.88	1.81	1.68	1.00	2.83
**Number of siblings**												
**Only child**	(reference)			(reference)			(reference)			(reference)		
**1**	**1.16**	**1.07**	**1.25**	1.06	0.95	1.17	**1.14**	**1.07**	**1.22**	**1.11**	**1.02**	**1.21**
**≥2**	**1.46**	**1.32**	**1.61**	**1.26**	**1.10**	**1.45**	**1.25**	**1.14**	**1.38**	**1.18**	**1.04**	**1.33**
**Equivalent income (million yen)**												
**<250**	(reference)			(reference)			(reference)			(reference)		
**250-399**	**0.89**	**0.81**	**0.98**	0.90	0.80	1.01	1.05	0.97	1.13	0.96	0.88	1.06
**400-549**	**0.76**	**0.68**	**0.84**	**0.76**	**0.66**	**0.87**	**1.26**	**1.15**	**1.39**	1.05	0.93	1.19
**550-699**	**0.80**	**0.71**	**0.91**	**0.74**	**0.62**	**0.88**	**1.33**	**1.15**	**1.53**	1.03	0.86	1.23
**700+**	1.01	0.88	1.16	**0.74**	**0.60**	**0.90**	**1.60**	**1.34**	**1.92**	1.17	0.94	1.47
**Living with grandparents**												
**No**	(reference)			(reference)			(reference)			(reference)		
**Yes**	**1.21**	**1.11**	**1.33**	**1.17**	**1.04**	**1.32**	0.98	0.90	1.07	**1.17**	**1.05**	**1.31**

Abbreviation: OR, odds ratio.

a Bold values denote statistical significance at *P* < .05.

b Adjusted model includes paternal work-related factors (paternal working hours per week, paternal commuting hours per day, paternal workplace headcount size) and covariates (father’s age at birth, mother’s age at birth, paternal education, maternal education, child sex, birth weight, child’s history of hospital visits for congenital diseases, child’s history of hospital admission for congenital diseases, number of siblings, equivalent income, living with grandparents).

None of the interaction terms between mother’s employment status and father’s work-related factors were significant (results are not shown). However, several covariates were differently associated with paternal outcomes depending on whether the mother was working. For example, when mothers were working, the maternal age was not significantly associated with the amount of time fathers spent with their children on working days or frequency of paternal childcare. When mothers were not working, it was found that the older the mother, the less time fathers spent with their children on working days and the lower the frequency of paternal childcare. Furthermore, when the mother was working, paternal higher education lowered the OR of spending less time with the child on nonworking days and also the OR of infrequent childcare. Household income was not significantly associated with paternal time-spending with children on working days, whereas the ORs of lesser paternal time-spending with children on nonworking days and less frequent childcare were lower in the higher income group. Conversely, when the mother was not working, the ORs of fathers spending less time with their children on working days increased with higher household income; and household income was related to neither paternal time spent with their children on nonworking days nor the paternal frequency of childcare.

## 4. Discussion

This study examined how paternal work-related factors, such as working and commuting hours and the number of employees at the fathers’ workplace, are associated with paternal involvement in childcare for toddlers in Japan. Our results also showed that fathers’ working hours far exceeded the legal working hours in Japan (40 hours per week), and longer commutes reduced the time fathers spent with their children on working days. Similarly, fathers’ longer working hours and commuting times were also associated with less frequent childcare, suggesting that longer work-related hours on working days may inhibit their time spent on activities at home. Similar results were observed regardless of whether the mother was working. In other words, regardless of whether the mother is working or not, and shares responsibility for childcare with the father, paternal work-related hours on working days can still have a significant impact on fathers’ involvement in childcare.

With regard to “nonworking days,” the association with fathers’ working hours was similar to that observed for working days; fathers who worked longer hours on working days spent less time with children. Conversely, fathers with longer commuting hours spent longer time with their children on “nonworking days” than those with less than 0.5 commuting hours. The mechanism behind these results may be that the length of their working hours reflects fathers’ intentions, but it is difficult for them to change commuting time according to their intentions. In other words, fathers whose long commuting hours prevent their involvement in childcare during the week might be more conscious of spending more time with their children on nonworking days or they may share the role of childcare on nonworking days with their partners.^8^^,^^20^ Fathers who work for more than 50 hours on working days per week may include those who choose to work longer hours themselves and are not actively engaged in parenting, even on nonworking days. Our results also show that fathers in the lowest working hours group (<20 hours per week, 4.2%) were less involved in childcare on both working days and nonworking days. Fathers who worked 20-39 hours per week, particularly on nonworking days and in families where mothers were not working, exhibited a higher OR of being less involved in childcare than fathers working 40-49 hours weekly. Fathers with fewer working hours may not be able to work for health or other specific reasons and thus may not be involved in childcare.

Generally, a larger company is considered to have more supportive accommodation for employees raising children because it has more resources to cope with their leave-taking and time arrangements (eg, hiring substitutes). Additionally, larger firms may be under more pressure to maintain social legitimacy by responding to recent policies that support fatherhood.^24^ However, previous studies conducted in other countries have reported inconsistent findings regarding the association between company size and paternity leave. Research in the United Kingdom, Luxembourg, and Australia has reported that larger companies are more supportive of taking paternity leave.^25-27^ However, the majority of large companies in Sweden, one of the first nations to offer fathers paid parental leave, were not supportive of taking paternal leave as of 2006.^28^ According to a previous study in Japan, fathers working in large companies reported working in a more father-friendly workplace than those working in small or medium-sized companies.^7^ Compared with fathers in workplaces with fewer than 100 employees (many of whom may be from smaller companies), those in workplaces with more than 500 employees spent less time with their children during the week and had less frequent childcare. These recent Japanese studies indicate that, although legally encouraged systems, including paternity leave, may have gradually become easier to avail in large companies in Japan, other factors related to daily operations, such as lack of flexibility in work hours, corporate culture for employees with children, and high levels of stress at work, could contribute to less time and frequency of paternal childcare. Alternatively, based on theories regarding the determinants of the division of household labor, it could be deduced that relatively higher resources (income, education, etc) or the ideology of the gender roles of fathers who work for large firms may clarify the division of domestic roles between couples.^29-31^

Our results also show that the effects of several parental factors (eg, mother’s age or father’s education) and household income on the father’s outcomes related to childcare differed by the employment status of the mother. These results indicate that couples make choices about how to commit to work and childcare based on their preferences and role divisions. However, regardless of the employment status of mothers, the effect of fathers’ work-related time on childcare-related outcomes may be crucial.

This study had some limitations. Fathers’ time of involvement in childcare was mostly answered by mothers, which may not describe the actual situation. The quality of marital relationships and maternal mental health are likely to bias the responses.^32^^,^^33^ However, we were unable to adjust for the quality of marital relationships or parental mental health. This study examined working hours, commuting time, and the number of employees as fathers’ work-related factors. However, to further clarify the relationship between fathers’ work style and childcare, future research should also consider job types and employment types. As this was a cross-sectional study, we were unable to examine the causal relationship between fathers’ longer work-related times and shorter working day parenting times. For example, a couple’s preferences regarding how to divide work and childcare may alter the relationship between work-related time and childcare. In the future, the impact of work-related time could be clarified if such preferences regarding the division of work and childcare among couples are considered. For fathers who wish to be actively involved in childcare, further discussions are required to determine how long working hours should be optimized to provide sufficient time for childcare.

Long working hours for Japanese fathers have been noted as a negative spillover into their role at home.^34^ The ideology of the Japanese ideal father may have changed in recent years in response to social conditions such as the advancement of women in society and the transition of the paternity leave system^34^^,^^35^; however, even if Japanese fathers aspire to a family-oriented fatherhood, they could still be forced to work long hours in the existing corporate culture due to their economic necessity, which would hinder their efforts. The study’s findings, in which social contexts such as working hours and workplace size affected parenting time, indicate that effective implementation of childcare support measures may not only depend on effective legal arrangements or raising awareness for individual fathers but also on approaches to fathers’ work styles and work environments.

## 5. Conclusions

In Japan, fathers who worked longer than the legal working hours or had longer commuting times were at a significantly higher risk of spending less time with their children on working days and were also less likely to frequently participate in childcare. Furthermore, these associations may be independent of the employment status of the mother. With the increasing number of options for working and involvement in child-rearing among fathers and mothers, the current situation in which fathers’ work-related hours interfere with their time at home will need to be changed to realize their desire for more active involvement in childcare.

## Data Availability

The data underlying this article were provided by the Ministry of Health, Labor, and Welfare in Japan by permission. Data cannot be shared on request.
